# Supplementary figures and images for: Individual-based multiple-unit dissimilarity: novel indices and null model for assessing temporal variability in community composition
Source: Oecologia. 2021 Sep 21;197(2):353–64. doi: 10.1007/s00442-021-05025-3 (PMC8505320; doi:10.1007/s00442-021-05025-3)

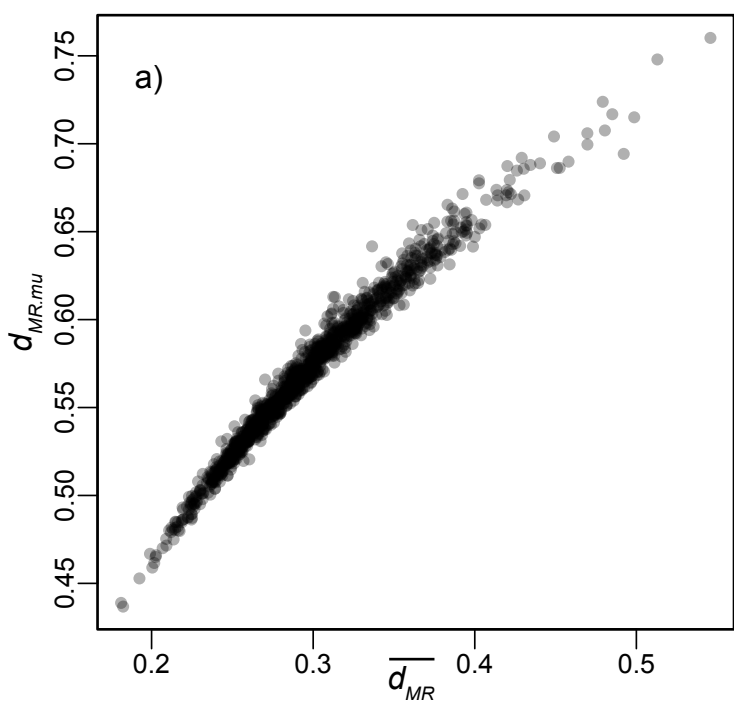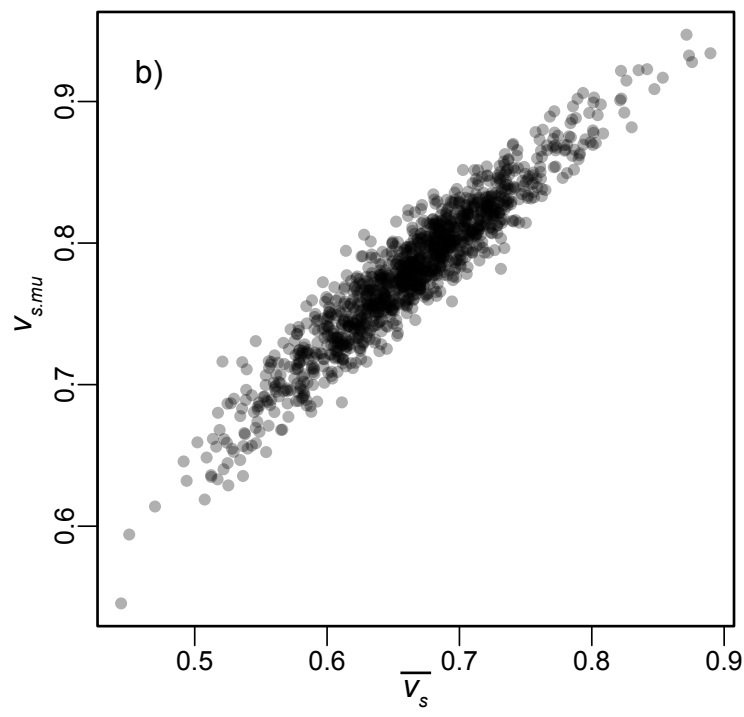

Supplement: Supplementary file 1 — Supplementary file1 (PDF 5154 KB) [file 442_2021_5025_MOESM1_ESM.pdf]

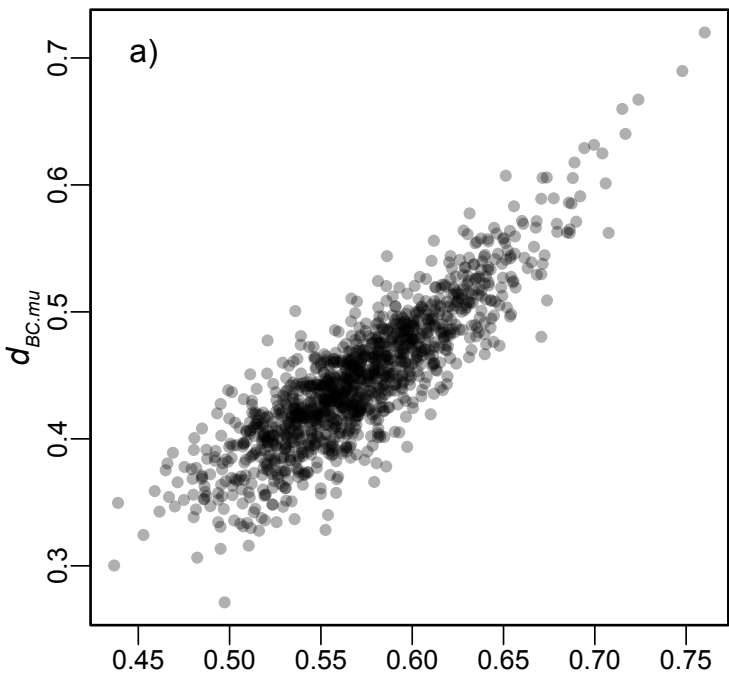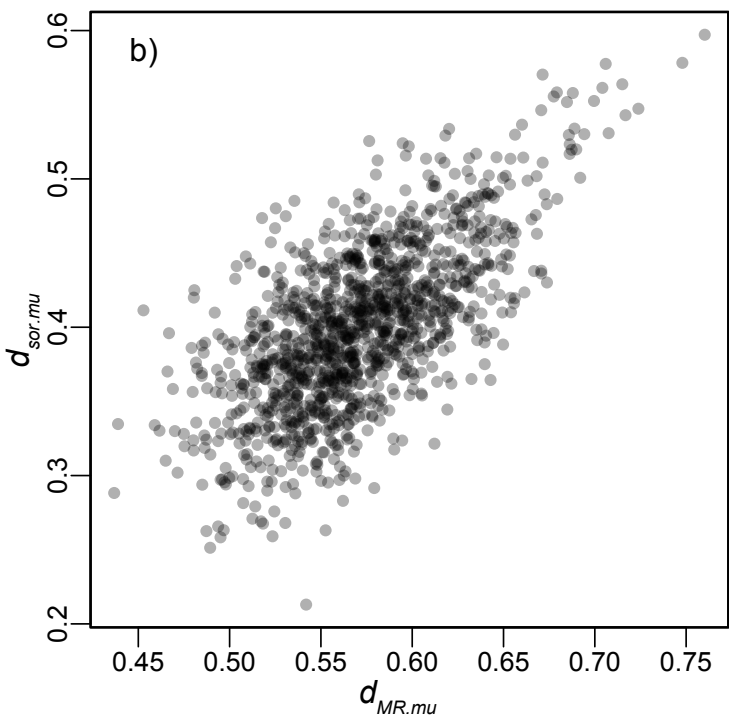

Supplement: Supplementary file 2 — Supplementary file2 (PDF 4897 KB) [file 442_2021_5025_MOESM2_ESM.pdf]

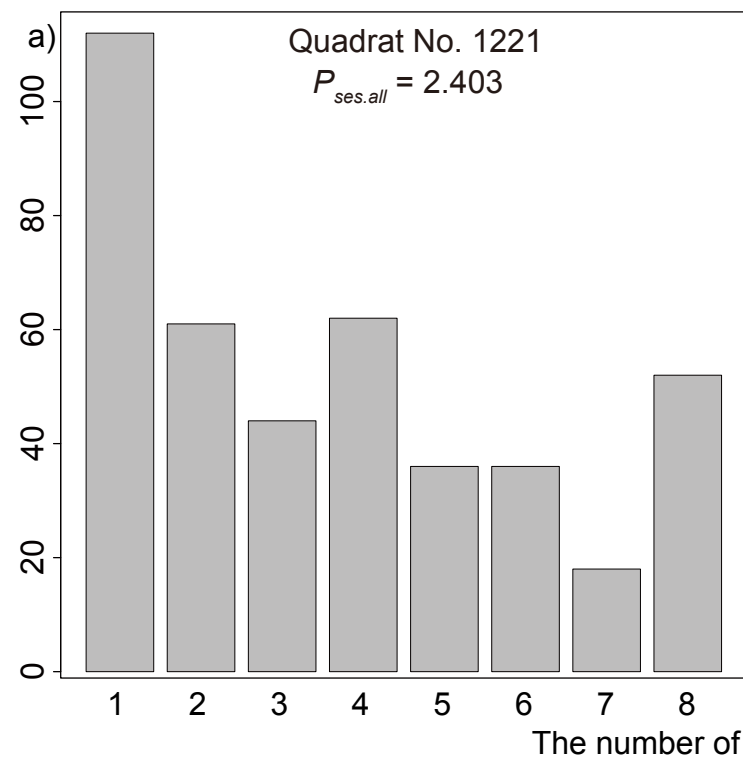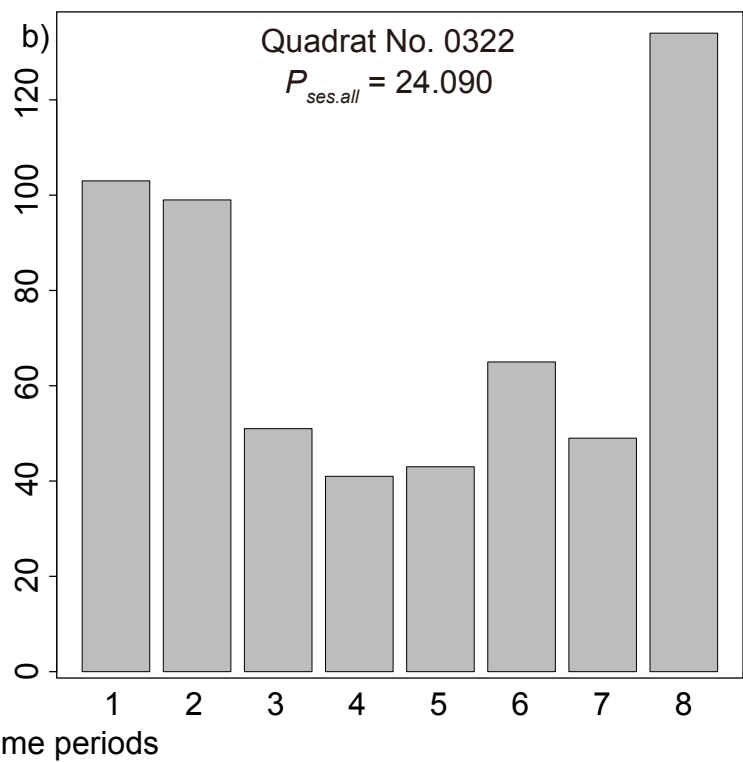

Supplement: Supplementary file 3 — Supplementary file3 (PDF 105 KB) [file 442_2021_5025_MOESM3_ESM.pdf]
